# Intravenous insulin therapy during lung resection does not affect lung function or surfactant proteins

**DOI:** 10.1186/1471-2466-14-155

**Published:** 2014-10-02

**Authors:** Zdenek Ručka, Irena Koutná, Lenka Tesařová, Michaela Potěšilová, Stanislav Stejskal, Pavel Šimara, Petr Vaňhara, Jan Doležel, Vaclav Zvoníček, Oldřich Coufal, Ivan Čapov

**Affiliations:** Masaryk University, Faculty of Informatics, Centre for Biomedical Image Analysis, Botanická 68a, 60200 Brno, Czech Republic; Faculty of Medicine, Department of Histology and Embryology, Masaryk University, Kamenice 3, 625 00 Brno, Czech Republic; 1st Department of Surgery, St. Anne’s University Hospital Brno, Pekařská 53, 656 91 Brno, Czech Republic; Department of Anaesthesiology and Intensive Care, St. Anne’s University Hospital Brno, Pekařská 53, 656 91 Brno, Czech Republic; Department of Surgical Oncology, Masaryk Memorial Cancer Institute, Zluty kopec 7, 656 53 Brno, the Czech Republic

## Abstract

**Background:**

The surgical resection of lung disrupts glucose homeostasis and causes hyperglycemia, as in any other major surgery or critical illness. We performed a prospective study where we carefully lowered hyperglycemia by insulin administration during the surgery, and for the first time we monitored immediate insulin effects on lung physiology and gene transcription.

**Methods:**

The levels of blood gases (pH, pCO_2_, pO_2_, HCO_3-_, HCO_3-_ std, base excess, FiO_2_, and pO_2_/FiO_2_) were measured at the beginning of surgery, at the end of surgery, and two hours after. Samples of healthy lung tissue surrounding the tumour were obtained during the surgery, anonymized and sent for subsequent blinded qPCR analysis (mRNA levels of surfactant proteins A1, A2, B, C and D were measured). This study was done on a cohort of 64 patients who underwent lung resection. Patients were randomly divided, and half of them received insulin treatment during the surgery.

**Results:**

We demonstrated for the first time that insulin administered intravenously during lung resection does not affect levels of blood gases. Furthermore, it does not induce immediate changes in the expression of surfactant proteins.

**Conclusion:**

According to our observations, short insulin treatment applied intravenously during resection does not affect the quality of breathing.

## Background

Stress accompanying a major surgery or critical illness disrupts glucose homeostasis. This disruption leads to hyperglycemia which is associated with a higher mortality rate of patients with severe illness [1]. Patients may also suffer from hyperglycemia during lung resection [2]. The protocol of decreasing stress-induced hyperglycemia involves insulin; however, the effect of insulin therapy on lung tissue is not clearly understood. To unravel mechanism of insulin action, we mapped gene transcription affecting lung surfactant production in resected lung tissue.

In our previous study, we focused on the effects of insulin on genes associated with lung surfactant in cancer cell lines. However, the results obtained from different cell lines differed significantly. Therefore, we designed and tested a way of studying insulin effects on gene transcription in patient lungs [3]. This study includes performing the same experiment on a larger cohort of patients. However, this time, we did not analyze only gene transcription but also changes in lung physiology accompanying insulin administration were closely observed. Our goal was to clarify whether or not short-term intravenous insulin therapy affects lung function. For our purposes, the parameters of acid-base balance obtained from arterial blood were selected as markers of lung function, and the composition of lung surfactant was also assessed through the transcription levels of lung surfactant-associated genes [4–6].

Lung surfactant is a mixture of phospholipids and proteins. Its primary role is to lower surface tension, which prevents the alveoli from collapsing. Its secondary role is linked to innate immunity because some surfactant proteins bind to pathogens and stimulate their phagocytosis and lysis. Four proteins are associated with lung surfactant [7]. Although these proteins only comprise approximately 10% of the surfactant mass, they are essential for its function [8]. The levels of proteins associated with lung surfactant and levels of their RNA were studied together with levels of blood gases in order to describe the effects of intravenously supplied insulin during lung resection.

While performing insulin therapy, it is crucial to prevent hypoglycemia. Studies on intensive insulin therapy point out the danger of hypoglycemia that occurs in up to 5% of patients and poses a severe complication. Due to this possible threat, the benefits of insulin therapy have been questioned [9]. Hypoglycemia was found to be connected to the time patients spend on the protocol (the longer the therapy, the higher the chance of hypoglycemia) making the strict monitoring of glucose levels even more important during intensive insulin therapy [10–12]. The latest results show that maintaining glucose levels at 7.8-10.0 mmol/l rather than 4.4 to 6.1 mmol/l decreases the number of hypoglycemic patients while preserving the beneficial effects of insulin therapy [13, 14]. In our study, the risk of hypoglycemia was lower because we utilized short insulin therapy. Nevertheless, blood glucose levels were strictly observed in order to protect patients’ health while monitoring the effects of insulin therapy during lung resection. The benefits of insulin therapy in surgical intensive care units have also been described [15].

## Methods

### Patients

This study was performed on a cohort of 64 patients (Table 1). The patients were suffering from lung adenocarcinoma (28), squamous cell carcinoma (12), large cell carcinoma (4), other tumors (10), metastasis (5), or non-malignant disease (5). The partial pressure of O_2_ (pO_2_; standard 72-100 mmHg) and CO_2_ (pCO_2_; standard 35-46 mmHg), the arterial blood pH (standard 7.35-7.45), HCO_3-_ (standard 21-26 mmol/l), HCO_3-_ std (standard 23-27 mmol/l), fraction of inspired oxygen (FiO_2_), Horowitz index (paO_2_/FiO_2_; 350-450 mmHg), and base excess (BE; standard -2 to +2 mmol/l) were measured in a blood sample collected anaerobically from peripheral artery [16–18]. The following types of lung resection were performed: lobectomy (53), pneumonectomy (2), and atypical resection (9). Surgery was performed under general anesthesia with added epidural anaesthesia or paravertebral block. The lung was ventilated selectively: pressure operated ventilation with FiO_2_ 0.4-0.7, positive end-expiratory pressure (PEEP) 5 cm, and inspire pressure 12-18 cm above PEEP in such a way that the breath volume was approximately 6 ml/kg and the pCO_2_ was expired in a mixture of 4.0-4.5 kPa. None of the patients experienced post-operative respiratory insufficiency. The levels of blood gases were determined in arterial blood obtained before the beginning of insulin administration (operation), at the end of the operation, and two hours after the end of the operation.

**Table 1:** **Basic characteristics of the total patient cohort**

	Total		Insulin treated		Control	
**Insulin treated**	**N**	**%**	**N**	**%**	**N**	**%**
Yes	31	48.4	31	100	-	-
No	33	51.6	-	-	33	100
**Age**	**Mean (SD)**	**Median (Min-Max)**	**Mean (SD)**	**Median (Min-Max)**	**Mean (SD)**	**Median (Min-Max)**
	64.9 (9.5)	66.2 (37.0-80.7)	65.0 (9.5)	68.3 (37.0-80.7)	64.7 (9.6)	64.9 (46.1-80.3)
**Sex**	**N**	**%**	**N**	**%**	**N**	**%**
Male	37	57.8	17	54.8	20	60.6
Female	27	42.2	14	45.2	13	39.4
**Diabetes**	**N**	**%**	**N**	**%**	**N**	**%**
No	49	76.6	20	64.5	29	87.9
Yes	15	23.4	11	35.5	4	12.1

The patient cohort was independently randomized into two groups: with or without insulin administration. Two subgroups of non-diabetic and diabetic patients were also distinguished. Insulin (insulinum humanum rapid) was administered to maintain glycemia at 6.0-8.0 mmol/l during surgery. The blood glucose levels were evaluated every hour in arterial blood. The average dose of insulin per patient was 24.15 units. Infusion of 10% glucose was administered. The speed of infusion was approximately 50 ml/h, and the glucose uptake was always maintained at 1 mg/kg/min. At the beginning of the study, six patients experienced hypoglycemia below 4 mmol/l; therefore, the first dosage of insulin was lowered from 8 to 6 units/h for patients who had normoglycemia before surgery. In concordance with the NICE-SUGAR study, glycemic levels were not maintained within 4.6-6.4 mmol/l range, but above 6.0 mmol/l [9, 19]. We kept it within the range of 6.0-8.0 mmol/l.

Samples from 46 patients were anonymized and analyzed by qPCR (Table 2); the other 18 samples had to be excluded from the qPCR analysis because of low RNA quality. A written informed consent form for the scientific use of biological material was signed by all the patients in accordance with the requirements of the St. Anne’s University Hospital Ethical Committee before the samples were included in the study.

**Table 2:** **Basic characteristics of the qPCR patient cohort**

	Total		Insulin treated		Control	
**Insulin treated**	**N**	**%**	**N**	**%**	**N**	**%**
Yes	23	50	23	100	-	-
No	23	50	-	-	23	100
**Age**	**Mean (SD)**	**Median (Min-Max)**	**Mean (SD)**	**Median (Min-Max)**	**Mean (SD)**	**Median (Min-Max)**
	65.0 (9.5)	66.2 (37.0-80.3)	64.7 (9.2)	66.1 (37.0-77.6)	65.3 (9.9)	66.4 (46.1-80.3)
**Sex**	**N**	**%**	**N**	**%**	**N**	**%**
Male	27	58.7	11	47.8	16	69.6
Female	19	41.3	12	52.2	7	30.4
**Diabetes**	**N**	**%**	**N**	**%**	**N**	**%**
No	36	78.3	15	65.2	21	91.3
Yes	10	21.7	8	34.8	2	8.7

### RNA isolation

After removal, samples of healthy lung tissue surrounding the tumor were immediately stored in RNAlater (Ambion, Austin, TX, USA) solution according to the manufacturer’s instructions until RNA was isolated. The samples were homogenized with a MagNA Lyser Instrument (Roche Diagnostics, Mannheim, Germany) prior to RNA isolation.

Upon homogenization, total RNA was isolated with the RNeasy kit (Qiagen, Hilden, Germany) according to the standard protocol. The RNA concentration was determined spectrophotometrically (NanoDrop ND-1000, NanoDrop Technologies, Wilmington, DE, USA). RNA integrity was analyzed with a 2100 Bioanalyser (Agilent Technologies, Santa Clara, CA, USA). Samples with a RIN number higher than 5, which indicated good quality RNA, were used for further analysis [20].

### Reverse transcription

cDNA was synthesized from 2 μg RNA using SuperScript II reverse transcriptase (Invitrogen, Carlsbad, CA, USA) and oligo (dT) primers (Sigma-Aldrich, St. Louis, MO, USA), according to the manufacturer’s instructions. RNasin RNase Inhibitor (Promega, Mannheim, Germany) was added.

### qPCR

The TaqMan® Gene Expression Master Mix was used together with the TaqMan® Gene Expression Assays SFTPA1: Hs01921510_s1, SFTPA2: Hs00359837_m1, SFTPB: Hs00167036_m1, SFTPC: Hs00161628_m1, and SFTPD: Hs00358340_m1 (Applied Biosystems, Foster City, CA, USA). 100 ng of cDNA and Human GAPDH Endogenous Control (Applied Biosystems, Foster City, CA, USA) were added to each reaction. The choice of GAPDH as a reference gene was based on GAPDH having a stringent correlation in the lung cells and the least variation of the seven common reference genes [21]. The PCR was performed in a 96-well plates format (25 μl per well) on a 7500 Real-Time PCR System (Applied Biosystems, Foster City, CA, USA). The temperature settings were as follows: step 1 at 50°C for 2 minutes, step 2 at 95°C for 10 minutes, step 3 at 95°C for 15 seconds, and step 4 at 60°C for 1 minute. Steps 3 and 4 were repeated 40×. The expression data were calculated using the comparative ΔCt method as described elsewhere [22]. Only normalization to the housekeeping gene (GAPDH) was used.

### Statistical analysis

Basic descriptions of the patients and variables were performed using frequency tables and the following descriptive statistics: mean, standard deviation, median, minimum and maximum. To evaluate the difference in values between the initial examination and subsequent examinations, a paired t-test was used. For evaluating the difference in values between two independent groups of patients, a standard two-sample t-test was applied. The normal distribution of values was verified graphically. For a non-normal distribution of values, the Wilcoxon test was used for paired data; for comparing two groups of patients, the Mann-Whitney test was used. The relationship between two continuous variables was assessed by Spearman’s correlation coefficient (due to the presence of outliers in the use of insulin). For qPCR experiments, the results were assessed with a two-way ANOVA with insulin administration as an explanatory variable. The standard level for statistical significance, α = 0.05, was used.

## Results

### Effects of insulin on lung physiology

To define the effects of the surgery and insulin, blood gas levels were measured at the initial examination, at the end of the surgery, and two hours after the surgery. The change was calculated as a difference between the final and first variable. Negative values represent a decrease in the selected variable during the surgery (Table 3).

**Table 3:** **Changes between the initial examination and the examination at the end of the surgery**

All patients N = 64	Mean change	SD	Median change	Min	Max	p
pH	-0.03	0.07	-0.03	-0.16	0.19	**0.001**
pCO_2_	0.13	1.10	0.10	-5.10	2.20	0.359
pO_2_	-1.81	10.52	-1.60	-36.10	26.60	0.173
HCO_3-_	-0.58	1.79	-0.55	-4.10	6.10	**0.012**
HCO_3-_ std	-0.88	1.50	-0.65	-4.30	2.20	**<0.001**
BE	-1.20	1.75	-0.90	-5.80	1.60	**<0.001**
FiO_2_	-0.02	0.19	0.00	-0.60	0.50	0.461
pO_2_/FiO_2_	-9.00	126.45	-25.35	-268.50	366.00	0.570
**Insulin treated N = 31**	**Mean change**	**SD**	**Median change**	**Min**	**Max**	**p**
pH	-0.02	0.06	-0.02	-0.15	0.09	0.069
pCO_2_	0.06	0.81	0.10	-1.20	2.20	0.675
pO_2_	-2.01	7.72	-2.10	-25.80	12.50	0.158
HCO_3-_	-0.12	1.83	-0.30	-4.10	6.10	0.719
HCO_3-_ std	-0.53	1.39	-0.30	-3.60	2.20	**0.044**
BE	-0.77	1.52	-0.50	-4.60	1.60	**0.008**
FiO_2_	0.00	0.18	0.00	-0.40	0.50	0.937
pO_2_/FiO_2_	-21.38	99.68	-30.00	-204.00	162.75	0.242
**Control N = 33**	**Mean change**	**SD**	**Median change**	**Min**	**Max**	**p**
pH	-0.04	0.07	-0.04	-0.16	0.19	**0.005**
pCO_2_	0.19	1.32	0.40	-5.10	2.00	0.420
pO_2_	-1.62	12.73	-1.40	-36.10	26.60	0.469
HCO_3-_	-1.01	1.67	-1.10	-4.10	2.10	**0.001**
HCO_3-_ std	-1.22	1.54	-1.50	-4.30	2.20	**<0.001**
BE	-1.61	1.88	-1.70	-5.80	1.30	**<0.001**
FiO_2_	-0.03	0.19	0.00	-0.60	0.40	0.362
pO_2_/FiO_2_	2.55	147.98	0	-268.50	366.00	0.922

Bold numbers indicate P value<0.05.

Statistically significant decreases were found between the initial examination and the examination at the end of the surgery in pH, HCO_3-_, HCO_3-_ std, and BE within the entire group and within the control group. Within the insulin-treated group, only small decreases were detected in HCO_3-_ std and BE (Table 3). When the entire insulin-treated group was compared with the entire control group, we found a statistically significant decrease in HCO_3-_ (p = 0.046) between the initial examination and the examination at the end of the surgery (Table 3 describes only changes within each group), whilst the other variables remained unchanged.

Similarly, statistically significant decreases were found between the initial examination and the examination 2 hours after surgery in pH, pO_2_, pCO_2_, HCO_3-_, HCO_3-_ std, and BE within the entire group and the control group. Within the insulin-treated group, decreases in pO_2_, pCO_2_, HCO_3-_, HCO_3-_ std, and BE were detected (Table 4). When the insulin-treated group was compared with the control group, we found that there is no statistically significant change in the observed variables between the initial examination and the examination 2 hours after surgery.

**Table 4:** **Changes between the initial examination and the examination 2 hours after the surgery**

All patients N = 64	Mean change	SD	Median change	Min	Max	p
pH	0.02	0.07	0.02	-0.13	0.19	**0.019**
pCO_2_	-0.84	1.00	-0.85	-4.00	1.30	**<0.001**
pO_2_	-12.02	10.79	-11.05	-48.80	15.40	**<0.001**
HCO_3-_	-2.00	2.36	-2.25	-7.80	7.00	**<0.001**
HCO_3-_ std	-1.15	1.98	-1.05	-6.30	2.60	**<0.001**
BE	-1.48	2.36	-1.55	-7.70	5.90	**<0.001**
**Insulin treated N = 31**	**Mean change**	**SD**	**Median change**	**Min**	**Max**	**p**
pH	0.03	0.06	0.03	-0.10	0.17	**0.008**
pCO_2_	-0.80	0.76	-0.80	-2.40	0.80	**<0.001**
pO_2_	-12.52	9.53	-9.70	-37.20	3.50	**<0.001**
HCO_3-_	-1.59	1.98	-1.70	-5.30	2.50	**<0.001**
HCO_3-_ std	-0.77	1.73	-0.90	-4.40	2.10	**0.019**
BE	-1.11	1.92	-1.40	-5.90	2.90	**0.003**
**Control N = 33**	**Mean change**	**SD**	**Median change**	**Min**	**Max**	**p**
pH	0.01	0.08	0.00	-0.13	0.19	0.377
pCO_2_	-0.87	1.20	-1.00	-4.00	1.30	**<0.001**
pO_2_	-11.55	11.98	-11.30	-48.80	15.40	**<0.001**
HCO_3-_	-2.38	2.64	-2.70	-7.80	7.00	**<0.001**
HCO_3-_ std	-1.50	2.16	-1.50	-6.30	2.60	**<0.001**
BE	-1.83	2.69	-1.80	-7.70	5.90	**<0.001**

Table 5 summarizes the changes observed between the initial examination and the examination at the end of the surgery in insulin-treated patients with or without diabetes. No significant differences were observed between the diabetes and non-diabetes group. However, significant differences were observed between the initial examination and the examination 2 hours after the surgery in insulin-treated patients with or without diabetes (Table 6). Statistically significant decreases in all of the measured variables were found for non-diabetic patients. In the diabetic group, almost the same changes (increase in pH, decrease in all of the other variables) were measured, but the results were not statistically significant. This lack of significance might be due to the smaller size of the diabetes group. These results are in agreement with the results of the entire insulin-treated group.

**Table 5:** **Changes between the initial examination and the examination at the end of the surgery in insulin-treated diabetic and non-diabetic patients**

Insulin treated N = 31	Mean change	SD	Median change	Min	Max	p
pH	-0.02	0.06	-0.02	-0.15	0.09	0.069
pCO_2_	0.06	0.81	0.10	-1.20	2.20	0.675
pO_2_	-2.01	7.72	-2.10	-25.80	12.50	0.158
HCO_3-_	-0.12	1.83	-0.30	-4.10	6.10	0.719
HCO_3-_ std	-0.53	1.39	-0.30	-3.60	2.20	**0.044**
BE	-0.77	1.52	-0.50	-4.60	1.60	**0.008**
FiO_2_	0.00	0.18	0.00	-0.40	0.50	0.937
pO_2_/FiO_2_	-21.38	99.68	-30.00	-204.00	162.75	0.242
**Non-diabetes N = 20**	**Mean change**	**SD**	**Median change**	**Min**	**Max**	**p**
pH	-0.01	0.05	-0.01	-0.14	0.09	0.269
pCO_2_	0.01	0.76	-0.10	-1.20	1.60	0.930
pO_2_	-1.04	5.98	-1.40	-10.90	12.50	0.448
HCO_3-_	-0.22	1.89	-0.40	-4.10	6.10	0.617
HCO_3-_ std	-0.47	1.37	-0.30	-3.60	1.90	0.141
BE	-0.69	1.56	-0.40	-4.60	1.60	0.062
FiO_2_	-0.01	0.14	0.00	-0.40	0.15	0.655
pO_2_/FiO_2_	-12.98	84.60	-25.35	-184.58	162.75	0.500
**Diabetes N = 11**	**Mean change**	**SD**	**Median change**	**Min**	**Max**	**P**
pH	-0.03	0.07	-0.02	-0.15	0.08	0.162
pCO_2_	0.15	0.93	0.20	-1.00	2.20	0.614
pO_2_	-3.78	10.27	-3.70	-25.80	8.40	0.250
HCO_3-_	0.05	1.80	0.10	-3.20	2.80	0.922
HCO_3-_ std	-0.63	1.49	-0.80	-2.50	2.20	0.193
BE	-0.92	1.50	-0.50	-3.40	1.30	0.070
FiO_2_	0.02	0.25	0.00	-0.40	0.50	0.811
pO_2_/FiO_2_	-36.53	125.63	-63.75	-204.00	148.73	0.357

Bold numbers indicate P value <0.05.

**Table 6:** **Changes between the initial examination and the examination 2 hours after the surgery in insulin-treated diabetic and non-diabetic patients**

Insulin treated N = 31	Mean change	SD	Median change	Min	Max	P
pH	0.03	0.06	0.03	-0.10	0.17	**0.008**
pCO_2_	-0.80	0.76	-0.80	-2.40	0.80	**<0.001**
pO_2_	-12.52	9.53	-9.70	-37.20	3.50	**<0.001**
HCO_3-_	-1.59	1.98	-1.70	-5.30	2.50	**<0.001**
HCO_3-_ std	-0.77	1.73	-0.90	-4.40	2.10	**0.019**
BE	-1.11	1.92	-1.40	-5.90	2.90	**0.003**
**Non-diabetes N = 20**	**Mean change**	**SD**	**Median change**	**Min**	**Max**	**p**
pH	0.03	0.06	0.03	-0.10	0.13	**0.030**
pCO_2_	-0.90	0.70	-0.95	-2.10	0.10	**<0.001**
pO_2_	-12.64	7.92	-11.75	-29.20	2.80	**<0.001**
HCO_3-_	-1.77	2.05	-2.00	-4.70	2.50	**0.001**
HCO_3-_ std	-0.92	1.70	-1.05	-4.40	2.10	**0.026**
BE	-1.36	2.04	-1.30	-5.90	2.90	**0.008**
**Diabetes N = 11**	**Mean change**	**SD**	**Median change**	**Min**	**Max**	**p**
pH	0.03	0.07	0.02	-0.07	0.17	0.148
pCO_2_	-0.63	0.86	-0.70	-2.40	0.80	**0.036**
pO_2_	-12.30	12.37	-9.10	-37.20	3.50	**0.008**
HCO_3-_	-1.25	1.90	-1.20	-5.30	0.90	0.054
HCO_3-_ std	-0.51	1.83	-0.70	-4.10	1.80	0.379
BE	-0.66	1.67	-1.40	-3.40	2.00	0.218

Bold numbers indicate P value <0.05.

Post-operative respiratory insufficiency was not reported in any patient regardless of insulin administration. During hospitalization, typically lasting 6 to 10 days, no organ failure was reported.

### Effects of insulin on gene transcription

The effect of insulin was also studied on anonymized samples from healthy lung tissue surrounding the tumor obtained from patients during lung resection (Table 7). To obtain valid results, only samples with high-quality RNA were included in the qPCR analysis of lung surfactant-associated genes. Therefore, the effects of insulin on gene expression were studied in only 46 out of the 64 patients.

**Figure 1:**
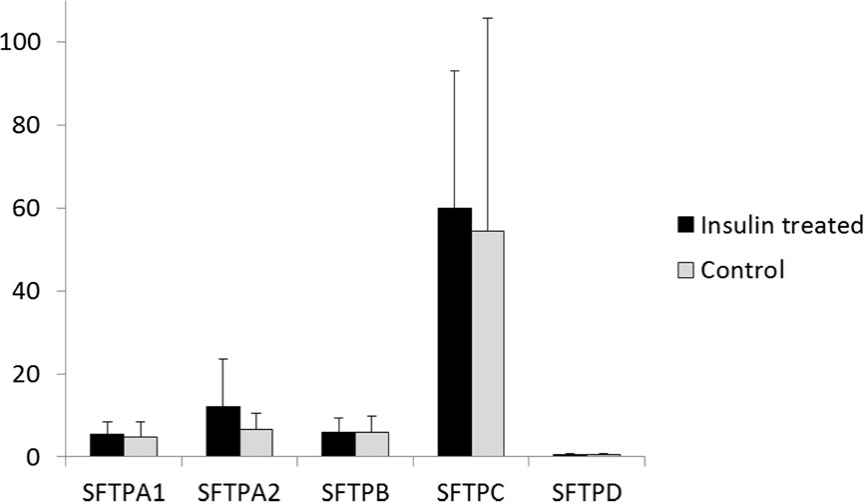
**Graph of selected gene expression levels normalised to GAPDH with and without insulin treatment.**

We demonstrated that expression levels of SFTPA1, SFTPA2, SFTPB, SFTPC, and SFTPD remain unchanged with respect to insulin treatment (Figure 1). Even though a distinct change was found in SFTPA2 expression level upon insulin treatment, it was not statistically significant (p = 0.095) (Figure 1).

**Table 7:** **Summary of selected gene expression levels according to insulin administration**

Gene	Insulin treated (N = 23)					Control (N = 23)					p
	Mean	SD	Median	Min	Max	Mean	SD	Median	Min	Max	
SFTPA1	5.44	3.04	5.40	0.75	13.13	4.59	3.73	2.95	0.54	15.24	0.130
SFTPA2	12.00	11.54	8.69	1.47	46.53	6.47	3.87	5.06	1.95	13.64	0.095
SFTPB	5.83	3.46	4.90	1.77	15.63	5.81	3.99	4.27	1.02	15.08	0.663
SFTPC	59.95	33.14	58.22	12.55	138.14	54.51	51.26	34.22	2.70	190.68	0.238
SFTPD	0.49	0.22	0.42	0.14	1.04	0.45	0.22	0.40	0.15	0.91	0.601

## Discussion

Our goal was to examine whether insulin affects the quality of breathing via affecting lung physiology and gene transcription. For our purposes, lung physiology was defined as blood gas levels and the genes of interest were the genes of surfactant proteins. The issue of insulin therapy and lowering hyperglycemia has been greatly discussed but to the best of our knowledge, there is no previous study focused on the immediate effects of insulin on lung physiology and the transcription of surfactant proteins performed on actual patients.

### Effects of insulin on lung physiology

Initially, we focused on the differences in blood gas levels among different groups of patients at different points in time (initial examination, end of the surgery, and 2 hours after the surgery). The comparison of the insulin-treated group and the control group showed that the only statistically significant difference was a decrease in HCO_3-_ (p = 0.046) between the initial examination and the examination at the end of the surgery. The paired t-test (p = 0.05) demonstrated no significant effect of insulin on oxygenation during lung resection (Table 3). When the group of insulin-treated patients was divided into non-diabetic and diabetic patients (Tables 5 and 6), the analysis showed no difference between these groups. However, it is important to keep in mind that the diabetes group was small; therefore, the statements based on the result of the diabetes group should not be overestimated.

If we focus on the changes within the selected groups (as shown in Tables 3, 4, 5 and 6) between the initial measurement and the end of surgery or two hours after surgery, we found the following: the results from the acid-base balance measurements represent almost typical data within the reference limits of all of the measured variables [23]. The mean data from the initial examination and the examination at the end of the surgery show low compensated respiratory acidosis in both groups – the control and the insulin-treated group [24]. This result seems to be partly caused by metabolic acidosis.

The evaluation of the differences between the initial examination, the examination at the end of the surgery, and the examination two hours after the surgery confirms a slight acidifying effect after the surgery (HCO_3-_, BE, and pH decreased, Tables 3 and 4). This effect was still present two hours after the surgery and it was most likely induced by stress caused by the surgery. Acidosis in the insulin-treated group was lower than the control group, but the difference was minimal and was most likely caused by the catabolic effect of insulin [25, 26].

Considering the partial pressure of oxygen, it must be noted that change of its value before and after surgery may be explained by many other conditions, for example: persisting anesthesia, atelectasis, bronchospasm, the extent of surgery, pain, and analgesia. Changes in the Horowitz Index (paO_2_/FiO_2_) were not significant.

Glycemia was also closely monitored during the experiment. As expected, our measurements revealed a significant difference in glycemia among the patients who were given insulin and those who were not (p = 0.002; data not shown). In the control group, the level of glycemia was increased. An increasing level of glycemia indicates the typical pathophysiological reaction to stress induced by surgery. This reaction was prevented by insulin administration. Unfortunately, there was a relatively high occurrence of hypoglycemia among the patients despite strict insulin regulation and the relatively short duration of the experiment (compared to intensive insulin therapy). In the insulin-treated group, there were 7 cases of hypoglycemia lower than 4 mmol/l and 1 case occurred in the control group. This result excludes the possibility that the lack of an effect of insulin on oxygenation could have been caused by an insufficient dosage of insulin during the experiments.

In summary, the statistically significantly lower level of acidosis in the insulin-treated group has no clinical value in the cohorts of patients included in this study. We demonstrated that intravenously supplied insulin during lung resection does not improve oxygenation.

### Effects of insulin on gene transcription

Based on our previous experiments on lung cancer cell lines [3] and other studies [5, 6], we anticipated that insulin therapy would regulate the expression of pulmonary surfactant-associated genes. However, qPCR analyses of lung tissue samples demonstrated that there are no statistically significant insulin-induced changes in the gene expression levels of SFTPA1, SFTPA2, SFTPB, SFTPC, and SFTPD upon insulin treatment. The level of SFTPA2 seems to double after insulin treatment, however the change was not statistically significant (p = 0.095). The probability that longer treatment or a higher dose of insulin could result in a significant change is low because results from other experiments suggest the opposite. Insulin is suspected to inhibit the transcription of SFTPA through the PI 3-kinase signaling pathway [6].

In human fetal lung explants maintained *in vitro* for 6 days, insulin was found to down-regulate SFTPA (isoforms were not distinguished) and SFTPB while not affecting SFTPC [4]. The isoforms of SFTPA not being distinguished in that study makes the comparison even more difficult.

In another study, the authors described the inhibitory effects of insulin on SFTPA (again the two isoforms were not distinguished) and SFTPB in the H441 cell line. The H441 cell line is used as a model of the Clara cells, lung surfactant-producing cells located in the bronchi. For SFTPA, a decrease was observed after only 4 hours of treatment, but it was 24 hours before an inhibitory effect was observed on SFTPB [27]. In our experiments, sample removal usually occurred 4 to 5 hours after the first administration of insulin to the patient. The effects of insulin were studied in another study using human fetal lung explants and H441 cells [5]. Insulin was found to down-regulate both SFTPA1 and SFTPA2 in fetal lung explants (cell culture was exposed to 2500 ng/ml insulin for 6 days) and SFTPA1 only in H441 cells (cell culture was exposed to 2500 ng/ml insulin for 24 hours).

Furthermore, in our own previously published experiments, the inhibitory effects of insulin on the transcription of the SFTPB, SFTPC and SFTPD genes in H441 cells and the SFTPB gene in A549 cells were observed [3].

In the situation in which insulin affects lung surfactant-associated genes differently in different models, we present results obtained from actual patients. The experiments performed with the cell lines may not accurately reflect the *in vivo* situation. Also, large changes in surfactant production and composition that occur in fetal lungs [28] cannot be reliably applied to mature lungs. We cannot exclude the possible effects of insulin on lung surfactant-associated gene expression after a longer period of time. However, insulin administration could not be prolonged in our experiments because maintaining stable condition of the patients was the priority. Administering insulin after the surgery would increase the risk of hypoglycemia and taking more lung samples after the surgery would be unacceptable.

## Conclusions

We demonstrated for the first time that insulin-induced changes in levels of blood gases and the parameters of acid-base balance obtained from arterial blood were minimal, and their physiological impact on patients was negligible.

Furthermore, gene expression analysis revealed no immediate changes induced by short-time insulin treatment in SFTPA1, SFTPA2, SFTPB, SFTPC, or SFTPD. Therefore, we conclude that according to our observations, intravenously supplied insulin during lung resection does not affect the quality of breathing. The other potential effects of insulin therapy and controlling glycemia were not the focus of this study. Based on the occurrence of hypoglycemia in our study, we also recommend maintaining glycemia levels within 6.0-8.0 mmol/l or within 7.8-10.0 mmol/l instead of the 4.6-6.4 mmol/l range. The first dose of insulin should not exceed 6 units of insulin per hour.
